# Identification of T-DNA structure and insertion site in transgenic crops using targeted capture sequencing

**DOI:** 10.3389/fpls.2023.1156665

**Published:** 2023-07-12

**Authors:** Eric Maina Magembe, Hui Li, Ali Taheri, Suping Zhou, Marc Ghislain

**Affiliations:** ^1^ Potato Agri-food Systems Program, International Potato Center, Nairobi, Kenya; ^2^ Department of Agricultural and Environmental Sciences, College of Agriculture, Tennessee State University, Nashville, TN, United States

**Keywords:** target capture sequencing, T-DNA insertions, transgenic events, regulation of GM crops, genetically engineered crops.

## Abstract

The commercialization of GE crops requires a rigorous safety assessment, which includes a precise DNA level characterization of inserted T-DNA. In the past, several strategies have been developed for identifying T-DNA insertion sites including, Southern blot and different PCR-based methods. However, these methods are often challenging to scale up for screening of dozens of transgenic events and for crops with complex genomes, like potato. Here, we report using target capture sequencing (TCS) to characterize the T-DNA structure and insertion sites of 34 transgenic events in potato. This T-DNA is an 18 kb fragment between left and right borders and carries three resistance (R) genes (*RB*, *Rpi-blb2* and *Rpi-vnt1.1* genes) that result in complete resistance to late blight disease. Using TCS, we obtained a high sequence read coverage within the T-DNA and junction regions. We identified the T-DNA breakpoints on either ends for 85% of the transgenic events. About 74% of the transgenic events had their T-DNA with 3*R* gene sequences intact. The flanking sequences of the T-DNA were from the potato genome for half of the transgenic events, and about a third (11) of the transgenic events have a single T-DNA insertion mapped into the potato genome, of which five events do not interrupt an existing potato gene. The TCS results were confirmed using PCR and Sanger sequencing for 6 of the best transgenic events representing 20% of the transgenic events suitable for regulatory approval. These results demonstrate the wide applicability of TCS for the precise T-DNA insertion characterization in transgenic crops.

## Introduction

Crop improvement is increasingly using genetic engineering to transfer genes not accessible by crossing or staking genes, which would require extensive backcrossing. Using the natural DNA transfer properties of *Agrobacterium tumefaciens*, a transfer DNA (T-DNA) bearing genes of interest is inserted into the plant genome (Hoekema et al., 1983; Gelvin, 2017; Anand and Jones, 2018). Ongoing efforts are to improve the transformation technology and expand its use to multiple crop species and genotypes (Anand and Jones, 2018). The use of *Agrobacterium*-mediated transformation is expected to increase by targeting T-DNA to specific genomic locations using genome editing technologies and delivering genome editing reagents to the plant cells (Sardesai and Subramanyam, 2018; Lee et al., 2019). These genetically engineered (GE) or transgenic plants must undergo safety assessments before approval for commercial release (Codex Alimentarius Commission, 2003). One of the principal criteria assessed is the molecular characterization of the T-DNA insertion in the plant genome, referred to as a transgenic event, which is described by the number of inserted copies, their integrity, and the flanking sequences in the host genome (World Health Organization, 2009).

The soil bacterium *A. tumefaciens* can transfer part of its tumor-inducing (Ti) plasmid, the T-DNA, to plants (Hooykaas and Beijersbergen, 1994). Virulence (*vir*) genes located on the tumor-inducing (Ti) plasmid control this transfer to the plant cell. The transfer system encoded by the *vir* genes resembles bacterial conjugation (Lessl and Lanka, 1994). However, some Vir proteins enter plant cells during T-DNA transfer to protect the T-DNA and mediate its transport to the nucleus (Citovsky et al., 1992; Regensburg-Tuink and Hooykaas, 1993; Rossi et al., 1996). The T-DNA is integrated into the plant genome by illegitimate recombination (IR), a mechanism that joins two DNA molecules that do not share extensive sequence homology, in this case, the plant DNA and T-DNA. In higher eukaryotic organisms such as plants, IR is the predominant mechanism of DNA integration (Offringa et al., 1990; Paszkowski and Scheid, 1998). The integration of the T-DNA relies mainly on DNA repair pathways in the host cell, and double-strand breaks (DSBs) are reported to be the preferred site for T-DNA integration (Salomon and Puchta, 1998; Chilton and Que, 2003; Tzfira et al., 2003; Kleinboelting et al., 2015). Recombination between several T-DNAs or fragments of them in different orientations occurs, and several concurrent integration pathways may underlie transformation events (Gelvin, 2017). The integration of the ssDNA, the T strand, of the T-DNA into the plant genome seems to be facilitated by host proteins acting at the 3’ and 5’ ends during DSB repairs (Van Kregten et al., 2016; Kralemann et al., 2022). These recent findings confirm T-DNA insertion is mediated by the own plant DNA repair machinery.

The molecular characterization of the T-DNA insertion pursues the general objective of providing information on the following intended and, possibly, unintended effects of the plant transformation (OECD, 2010). First, there must be at least one intact copy of each transgene inserted into the plant genome for them to be functional. Secondly, the T-DNA insertion may have interrupted an important gene resulting in undesirable characteristics. Thirdly, concatenated copies of a T-DNA, or parts of it, can alter transgene expression by co-suppression (Wang et al., 2012). Fourthly, the presence of backbone vector sequences is not recommended by most regulatory agencies, especially if they contain bacterial genes. Moreover, the junction sequence between the T-DNA and the plant genome could create new open reading frames that express new proteins or peptides whose toxicity and allergenicity potential must be assessed. It should be noted that the flanking sequence information helps to develop event-specific diagnostic assays to monitor, trace and manage the transgenic variety upon commercialization. It can also be used to trace the introgression of transgenic loci into breeding populations (Li et al., 2019).

The T-DNA structure and junctions can be analyzed by sequencing across the integrated T-DNA extending into host-plant-DNA junction. This sequence information can be used to infer the length of integrated T-DNA, whether or not the plasmid DNA backbone integration occurred, or whether the T-DNA is truncated or rearranged, which may lead to transgene inactivation or silencing (Kumar and Fladung, 2001; Kumar and Fladung, 2002; Somers and Makarevitch, 2004; Zeng et al., 2010). Insertion of the T-DNA can also occur in the form of multiple concatenated full and partial fragments that sometimes lead to intra- and inter-chromosomal rearrangements (Jupe et al., 2019). The sequences flanking the T-DNA insertion reveal the precise insertion site in the plant genome. This can also reveal whether the T-DNA insertion has led to deletions or rearrangements of plant genome sequences, as well as the interruption of existing genes or the presence of transposable elements at the site of integration (Bartlett et al., 2014). It is important to note that the genome structure at sites of T-DNA insertion or the structure of the T-DNA insertion itself can induce epigenetic alterations with detrimental effects on transgene function (Jupe et al., 2019).

Several strategies, referred to as conventional here, have been used for identifying T-DNA insertion sites, including Southern blot analysis, quantitative PCR (qPCR; Ingham et al., 2001), digital droplet PCR (Glowacka et al., 2016) and plasmid rescue (Nan and Walbot, 2009). Other methods for mapping T-DNA insertions are primarily PCR-based, including thermal asymmetric interlaced PCR (TAIL-PCR) (Liu et al., 1995; Singer and Burke, 2003; Liu and Chen, 2007), adaptor PCR, sometimes referred to as anchored PCR (Singer and Burke, 2003; Thole et al., 2009), and T-linker PCR that utilizes a specific T/A ligation (Yuanxin et al., 2003). These conventional strategies are all time-consuming, and their abilities to fully characterize T-DNA insertions are limited by various factors, including complex insertion patterns, T-DNA rearrangement, small insertions/deletions and individual nucleotide substitutions (Guo et al., 2016). In addition, it is often challenging to scale these methods up for high throughput screening (Ji and Braam, 2010). Even though these methods have been successfully used for the identification of the insertion site and flanking sequence in many GE crops (Windels et al., 2001; Yang et al., 2005; Akritidis et al., 2008; Bodi et al., 2013 Fraiture et al., 2015), their applicability in transgenic events with complex genomes is often unsatisfactory (Siddique et al., 2019).

Next-generation sequencing (NGS) can be used to map T-DNA insertions in plants, depending on its sequencing depth (Polko et al., 2012; Lepage et al., 2013; Inagaki et al., 2015; Zastrow-Hayes et al., 2015; Giolai et al., 2016; Guo et al., 2016). Kovalic et al. (2012) reported ~70x coverage within junction regions with NGS. While it is possible to expand the coverage of the genome using whole genome sequencing (WGS), this increases the cost of molecular characterization, which can be significant when assessing multiple transgenic events from polyploid crops like potato to select the most suitable for regulatory approval prior to environmental and commercial release. Clearly the application of WGS for molecular characterization of transgenic crops may be too expensive for companies or institutions with modest budgets that need to cover the high cost of experiments, resources needed to conduct extensive bioinformatics data processing, and purchase/maintain storage space for the massive amount of NGS data they generate (Nekrutenko and Taylor, 2012). Zastrow-Hayes et al. (2015) demonstrated the use of the plasmid sequence capture method coupled with NGS technology for sorting transgenic events for regulatory approvals. Their method referred to as Southern-by-Sequencing was confirmed to reach the same conclusion as those obtained from conventional methods (Brink et al., 2019). Those NGS methods are also suitable for the identification of unauthorized transgenic events (Wahler et al., 2013).

In this study, we used target capture sequencing (TCS) coupled with Illumina sequencing to identify and characterize the T-DNA insertion site and flanking sequences of 34 transgenic events in potato bioengineered for late blight (LB) disease resistance that have been shown to have complete resistance to LB in the field over several seasons (Ghislain et al., 2019; Webi et al., 2019; Byarugaba et al., 2021). Our method, though developed independently, is similar to the Southern-by-Sequencing method of Zastrow-Hayes et al. (2015). The molecular characterization needed for regulatory approval for environmental release is complicated by the relatively large genome size, the tetraploid nature of the transgenic varieties, and the presence of numerous homologs of the three resistance (*R*) genes from wild relatives [*RB*, *Rpi‐blb2* from *Solanum bulbocastanum* and *Rpi‐vnt1.1* from *S. venturii*] present in these transgenic events. These features make the characterization of T-DNA insertion sites using conventional methods more challenging. Previous studies in other GE crops such as GE Maize (Siddique et al., 2019), GE rice (Yang et al., 2013) and GE soybean (Kovalic et al., 2012) indicated that combining NGS with PCR validation could be a solution for the molecular characterization of GE potato. Alternatively, TCS is expected to increase the coverage within T-DNA and junction regions without generating a large amount of host genomic sequences. Thus, the number of copies of inserted T-DNA, their integrity, and their precise position on the potato genomic map could be inferred. This would, in a single step, provide the detailed characterization of T-DNA insertion in transgenic events required for regulatory approvals for future environmental and commercial release.

## Materials and methods

### Plant materials

Transgenic events were obtained from varieties grown widely in East and Central Africa (‘Desiree’, ‘Victoria’, ‘Tigoni’ and ‘Shangi’) by *Agrobacterium*-mediated transformation using the pCIP99 binary vector (https://www.ncbi.nlm.nih.gov/nuccore/MN164628) carrying three resistance (*R*) genes – *RB*, *Rpi-blb2* and *Rpi-vnt1.1 –* previously cloned from the wild potato relatives *Solanum bulbocastanum* and *S. venturii.* These transgenic events were genotyped earlier using Southern blot analysis to establish that they had a single T-DNA insertion and had no large fragments of the backbone vector sequence, but their precise T-DNA structure and insertion site were unknown (Ghislain et al., 2019; Webi et al., 2019 and Byarugaba et al., 2021).

### DNA extraction

Total DNA was extracted from potato leaves of the transgenic events and their non-transgenic equivalents. 100 mg of plant material was placed in a 2 ml microcentrifuge tube with grinding beads, and the tissue was disrupted using the FastPrep 24 Homogenizer (MP Biomedicals). Genomic DNA was then extracted using the DNeasy Plant Mini Kit (Qiagen) based on the manufacturer’s recommendations. The quality of the extracted potato genomic DNA was evaluated by UV-spectrophotometry with a Nanodrop ND-8000 instrument (Thermo Fischer Scientific) and 1% agarose gel electrophoresis. DNA samples with A260/A280 ratio between 1.8 and 2.0 and A260/A230 ratio in the range of 2.0–2.2, displaying a thick high molecular weight band on gel electrophoresis, were considered as good enough quality for genomic library preparation.

### NGS library prep, targeted capture enrichment and sequencing

DNA from each transgenic potato event was quantified by the Qubit™ 3.0 Fluorometer (Thermo Fisher Scientific). DNA was then normalized to 1,000 ng and subjected to mechanical fragmentation using LE220 Focused-ultrasonicator (Covaris, Inc., Woburn, MA) with 105 Watt Peak Incident Power, 5% Duty Factor, 200 Cycles per burst, and 50 seconds Treatment Time. The shearing was optimized to shear DNA down to ~750 bp fragments. The length, quantity and quality of sheared double-stranded DNA was analyzed by Bioanalyzer 2100 High Sensitivity DNA chip (Agilent Technologies, Santa Clara, CA).

For NGS library prep, samples normalized to 200 ng of fragmented DNA were end-repaired, ligated with adapters (NEBNext Ultra II DNA Library Prep Kit for Illumina, cat. E7645, NEB, Ipswich, MA); dual-indexed with NEBNext multiplex oligos for Illumina set I & set II (New England Biolabs catalog no. E7334S and E7500S); size selected and cleaned up using Agencourt AMPure XP beads (cat. A63881, Beckman Coulter, Brea, CA).

For target capture probe design, considering the tetraploid nature of the potato and the low capture probe count via 1x tile design, we decided to utilize a 2x tiling strategy for increased target capture efficiency. This led us to a total probe count within a pool to 413 xGen Lockdown Probes (Integrated DNA Technologies, Inc) of 120 bp each with a 60 base overlap to cover uniformly the entire 18 Kb T-DNA region of pCIP99. xGen Lockdown Probes were individually synthesized, 5’-biotinylated oligos for target capture. One pool of xGen Lockdown Probes was cost-efficient as it was enough for all 34 transgenic events. The target enrichment was done by hybridization of the xGen Lockdown Probes to the pooled DNA library. Dynabeads M-270 Streptavidin (Life Technologies, Cat #65305) was used to bind hybridized target DNA fragments. Post capture PCR enrichment was performed using KAPA HiFi HotStart Ready Mix (Kapa Biosystems, MA) with Illumina P5 and P7 primers.

The final library was validated using Bioanalyzer 2100 High Sensitivity DNA chip (Agilent Technologies, Santa Clara, CA) and Qubit 2.0 Fluorometer High Sensitivity kit (Life technologies, Austin, TX) to check the size selection efficiency and to define the concentrations of libraries. The libraries were normalized to a 2nM sequencing stock concentration, denatured with NaOH to single strand, and diluted before being ready to load onto the sequencer.

The target enriched libraries were pooled, and a final 2 nM sequencing stock was prepared. Illumina MiSeq (V3) PE300 (Illumina, San Diego, CA) sequencing was performed on a single lane flow-cell following the manufacturer’s protocol at Vanderbilt Technologies for Advanced Genomics (https://www.vumc.org/vantage/home). The raw sequencing data were de-multiplexed based on the individual “barcode”/index added to each DNA sample during library preparation. FastQC filtering was applied to remove low quality reads of insufficient length. Trimmomatic was performed to remove the adapter sequences before data analysis.

### Sequence analysis

DNA sequence analysis and mapping of sequence reads to the reference sequence (T-DNA, vector, and potato genomes) were carried out using the CLC Bio genomics workbench version 8.0.3 and Geneious 9.0 software. The two software programs were used for analyzing the read quality and trimming ambiguous low-quality reads. After quality assessment and trimming, total cleaned reads were obtained with reads of 300 bases. The reads were mapped to the pCIP99 binary vector, including the T-DNA region as the reference sequence, and the resulting consensus sequences were scrutinized for differences with the reference sequence using both software programs. Using CLC Bio, up to 750 bp of mapped sequences were extracted at the left and right end of the T-DNA/flanking potato sequences using 90% sequence identity. Extracting all mapped reads and then assembling using CLC Bio increased the number of contigs too much to analyze, whereas increasing the % sequence identity reduced the number of assembled contigs. The consensus sequence of each of the contigs was aligned with the pCIP99 T-DNA to deduce the precise T-DNA/potato sequence junction. Using Geneious 9.0, the consensus sequence was extended on both ends separately by mapping reads to the T-DNA extended end repeatedly until no reads extended further than the consensus sequence, using 97% sequence identity. Since the goal of this study was to identify the insertion sites rather than SNP variants or expression variations, optical duplicates were not considered as a problem and no bioinformatic application was done to remove them.

Mapping of the flanking potato sequences (flanks) from each of the events was made using the potato reference genome v6.1 of the doubled monoploid *Solanum tuberosum* Group Phureja clone DM1-3 (DM) (Spud DB http://solanaceae.plantbiology.msu.edu/pgsc_download.shtml) and of the *Solanum tuberosum* cultivar *Solyntus* (Accession NC_008096 GenBank at NCBI).

### Validation of T-DNA/potato junction by PCR and resequencing

The T-DNA flanking region sequences of the transgenic events and the T-DNA end regions were used to design event-specific primers for each of the transgenic events with T-DNA insertions suitable for regulatory approval, in order to amplify T-DNA junctions (across the integrated T-DNA/host-plant DNA junction). Genomic DNA normalized to 10 ng/μl was then used for event-specific PCR analysis to validate and identify each event. The reaction was set up using the AccuPower ^®^PCR PreMix (Bioneer). Each reaction contained 1.0 μl of DNA (10 ng/μl), 0.5 μl each of the forward (5 μM) and reverse (5 μM) primers and 8.0 μl of nuclease free water for a 10 μl reaction. DNA from a non-transgenic plant was used as a negative control. The PCR reaction was done under the following conditions: 94˚C for 5 minutes followed by 35 cycles of 94˚C for 30 seconds, primer annealing temperature for each primer set for 30 seconds and 72˚C for 60 seconds then 72˚C for 10 minutes and 4˚C hold. 2% agarose gel electrophoresis was used for visualization of PCR products. The PCR products were purified and Sanger sequenced using BigDye Terminators Version 3.1 kit (Applied Biosystems) with the relevant primers on an ABI 3730 sequencer (Applied Biosystems).

## Results

### T-DNA targeted capture sequencing coverage

A total of 34 transgenic events from the varieties Desiree (2), Shangi (10), Tigoni (6), and Victoria (16) were successfully sequenced to characterize, at least partially, their T-DNA insertion site. Sequencing of the T-DNA enriched libraries produced paired-end reads ranging from 702,144 to 2,735,631 with an average of 1,413,131 reads per sample ( **Supplementary Table 1**).

**Figure 1:**
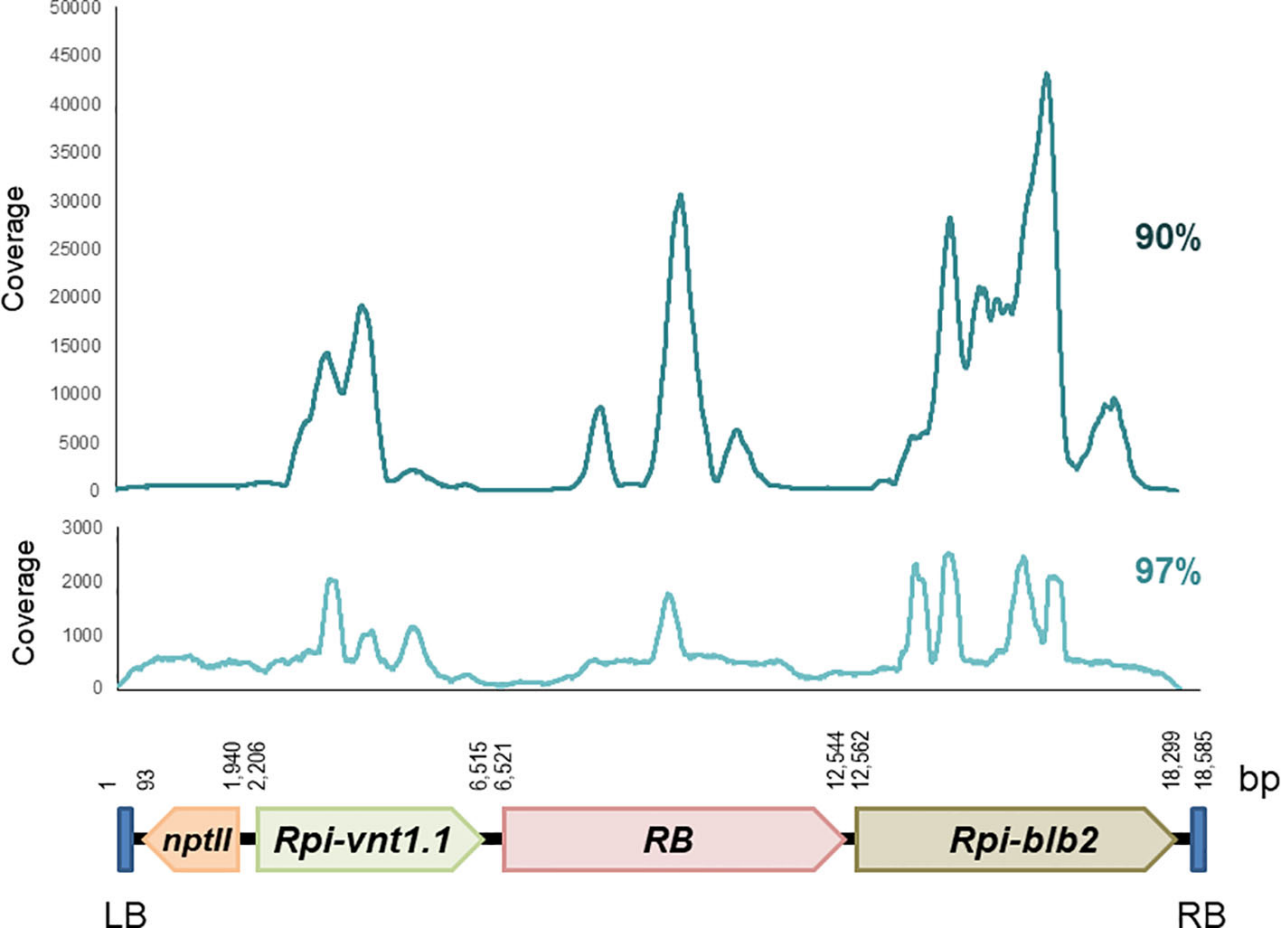
Coverage of paired reads mapped to the T-DNA at 90% and 97% sequence identity obtained from target capture sequencing of the potato transgenic event Vic.172.

Reads were mapped to the 18,585 bp T-DNA of the binary transformation vector pCIP99, which was used to produce the transgenic events. Mapping to the T-DNA reference was done at 90% and 97% sequence identity. We did not use 100% because reads have a sequencing error rate (average Q20 is 91%) (**Supplementary Table 1**). In all cases, coverage was enough to unambiguously identify the consensus sequence and compare it to the T-DNA of pCIP99. The CLC Bio and Geneious software gave very similar results. Coverage was uneven across the T-DNA sequence with specific regions of the potato *R* genes exhibiting high coverage due to the presence of multiple *R* gene homologs in the potato genome (**Figure 1**, **Supplementary Table 1**).

### T-DNA sequence breakpoints

To identify the junctions between the T-DNA and the potato genome sequences, the CLC Bio method (contig assembly from extracted end reads) and the Geneious method (iterative end extension by read mapping) gave identical breakpoints (T-DNA nucleotide next to first nucleotide of the flanking sequence). It is worth noting that the results of the breakpoint analyses are conditioned by two transgenic event pre-selection criteria. On both ends, transgenic events were pre-selected for the absence of large portions of the backbone vector sequences (bearing functional genetic elements) by PCR. Any transgenic event with a positive PCR amplification beyond the left and right border sequences into the backbone vector sequence, was eliminated and not included in the TCS analysis (Ghislain et al., 2019). Secondly, any left end T-DNA occurring within the *nptII* coding sequence might have altered the *nptII* gene to be non-functional and, thus, suppress the ability of the transgenic event to grow on media with kanamycin.

**Figure 2:**
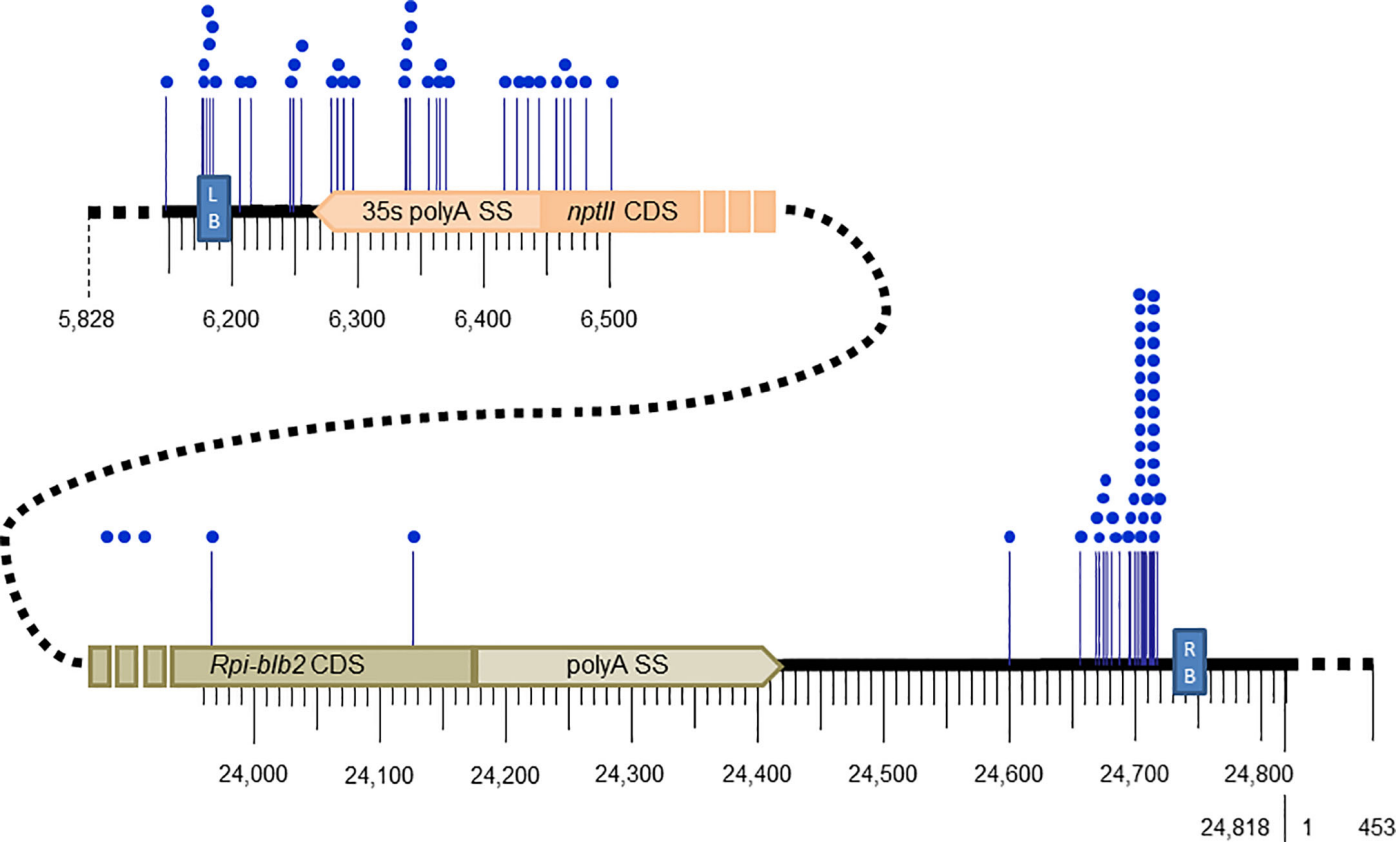
Breakpoint positions (blue dots) along the T-DNA of pCIP99 (24,818bp) at the junctions of the left and right end of the inserted T-DNA into potato transgenic events.

Out of the 34 transgenic events, the breakpoint of the left end T-DNA was identified in 30, with 4 transgenic events having 2 left breakpoints, for a total of 34 characterized left junctions (**Supplementary Table 2**). The recombination between the left end of the T-DNA and the potato genome appears to be localized randomly in a 330 bp region between the left border sequence and the *nptII* CDS (**Figure 2**, **Supplementary Table 2**). Integration beyond the left border was detected for 4 transgenic events: one transgenic event showed the integration of a 344 bp region beyond the LB until position 5,828, and 3 others had longer backbone vector sequences beyond the LB which were apparently misidentified as PCR negative when testing for the presence of backbone vector sequence (**Supplementary Table 2**).

Out of the 34 transgenic events, the breakpoint of the right end T-DNA was identified in 27, of which 5 have 2 to 5 right flanks totaling 44 characterized right flanks (**Supplementary Table 2**). Unlike for the left end T-DNA, 39 out of the 44 (89%) breakpoints in the right end T-DNA were localized in a small region of 60 bp downstream of the right border sequence by 15 bp, and within this region, 26 of the 39 breakpoints were localized in a region of 15 to 25 bp (**Figure 2**, **Supplementary Table 2**). No integration of vector sequence beyond the right border was detected.

### T-DNA sequence flank lengths

The length of the flanks varied when using either CLC Bio or Geneious software for the same genotype. Geneious provided on average longer flanks (**Supplementary Table 2**). The average flank length at the left end T-DNA was 344 bp in CLC Bio and 567 bp in Geneious. For the right end T-DNA, the average flank length was 301 bp using CLC Bio and 432 bp using Geneious. In addition, Geneious provided an easy visual identification of additional flanks representing additional T-DNA fragments inserted in tandem with the T-DNA or in another site. In those cases, it was not possible to identify the end of the additional T-DNA sequence due to the short size of Illumina reads. For these reasons, the results reported hereafter are those obtained using Geneious software. Flanking regions were not clearly identified for 2 or 3 transgenic events for the left flank and 7 for the right flank (**Supplementary Table 2**). Flanks were either potato genome (Tbr), T-DNA, or vector sequences and combinations thereof (**Supplementary Table 2**).

**Figure 3:**
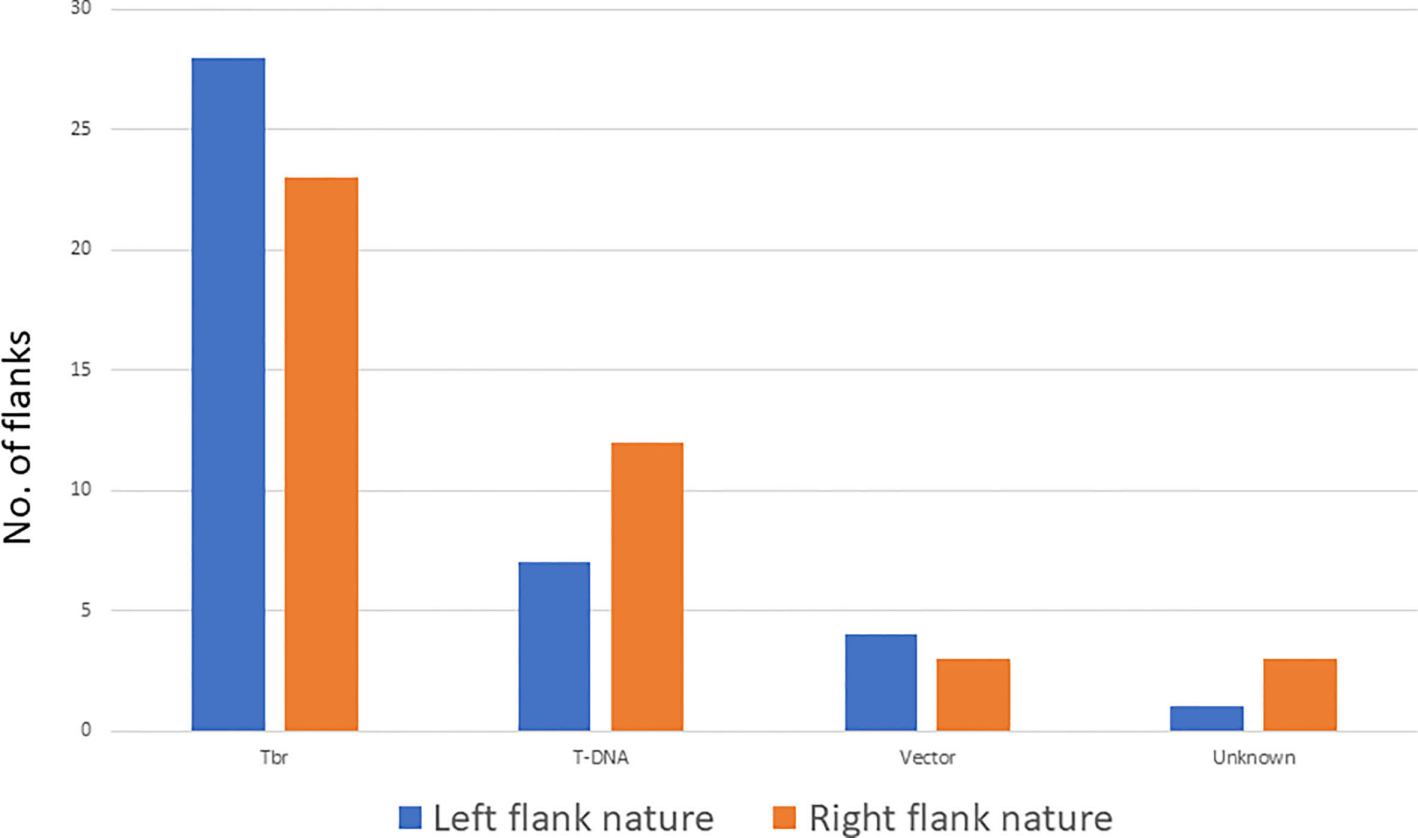
Nature of the flanking sequences of the T-DNA insertion on the left and right end. Tbr stands for potato genome.

Twenty-eight flanks on the left end of the T-DNA are potato genome flanks whereas seven are T-DNA sequences, 4 are vector sequence beyond the boarder, one is a combination of T-DNA with a potato genome sequence, and one is of unknown nature (**Figure 3**, **Supplementary Table 2**). For 3 of the left flanks, we identified a DNA sequence between the breakpoint and the identified flank (T-DNA, vector, or Tbr) of unknown identity. These sequences, referred to as filler sequences (Gheysen et al., 1991), ranged from 8 to 68 bp. Out of the 34 transgenic events, 4 had more than one left end. The short size of the Illumina reads did not allow to determine whether these additional ends were from additional inserted T-DNA sequences in the same loci or at different loci.

On the right border, twenty right flanks belong to the potato genome, eleven are T-DNA, 2 are vector sequences, three are combinations of T-DNA with potato genome sequence, 1 is a fragment of the Ti plasmid, and 4 are of unknown nature (**Figure 3**, **Supplementary Table 2**). Filler sequences were identified in 7 flanks ranging from 6 to 40 bp. Out of the 34 transgenic events, 7 had more than one right end. Only one of them had multiple left ends. Again here, full resolution of these ends were not possible due to the short size of Illumina reads.

### Validation of T-DNA/potato junction

The T-DNA and flanking sequences observed by bioinformatics analysis were validated by PCR for 6 transgenic events (Des.52, Vic.172, Vic.185, Tig.254, Sha.6 and Sha.105) in which T-DNA insertion was suitable for regulatory approval and no existing genes were interrupted. In addition, these junction sequences were used to design event-specific primers for each insertion site, followed by PCR amplification and Sanger sequencing to validate the accuracy of target capture sequencing. In each case, the deduced Sanger sequence confirmed the correct identification of the junction between the T-DNA and the potato genome. For those transgenic events that will be further tested in the field, and eventually deployed to farmers, the event-specific primers will be used for establishing event-specific detection tools.

### T-DNA sequence integrity

The consensus sequence was obtained from the reads that mapped to the T-DNA reference and was used to assess the DNA sequence integrity of the T-DNA of each transgenic event. Small insertions, deletions, single nucleotide polymorphisms, and rearrangements were looked at along the T-DNA. About 74% (25 out of 34) of the transgenic events have an intact copy of the T-DNA whereas two have a fragmented T-DNA. Five transgenic events have a truncated T-DNA on their right end within the *Rpi-blb2* genes, whereas two have either a SNP or a short deletion in their T-DNA (**Supplementary Table 2**). The remaining 9 transgenic events lack potential for regulatory approval due to DNA sequence changes implying functional alteration of at least one of the 3 *R* genes.

About 29% (10 of 34) of the transgenic events have one copy of the T-DNA with the complete and intact DNA sequence of the 3 *R* genes without additional T-DNA or vector fragments and with potato flanks on both sides (**Supplementary Table 2**). Of the remaining 15 that have an intact T-DNA, 10 have extra fragments of the T-DNA, which impeded full characterization of the insertion site; one transgenic event has a vector sequence whose recombination with the potato genome did not have coverage, 3 transgenic events have integration of vector sequence beyond the *aadA* bacterial gene, and one transgenic event has a Ti plasmid fragment including two bacterial genes (from the last third of the orf Bo191 Ti gene to another Ti gene orf Bo 192 of the Ti plasmid pTiBo542). The last 4 transgenic events disqualify as having potential for regulatory approval due to the presence of bacterial genes.

### T-DNA insertion site in the potato genome

The length of the flanks and the representation of the potato reference genomes have influenced the identification of the insertion site of the T-DNA. The mapping of the left and right flanks was conducted independently on the reference genome *S. tuberosum* Group Phureja DM1-3 and the sequenced potato genome *S. tuberosum* cultivar *Solyntus* (Accession NC_008096) but results were highly concordant (**Table 1**).

**Table 1:** T-DNA insertions in the potato genome: map position, deletion, and existing allele affected.

Transgenic event		Integration site in potato reference genome DM v6.1	Deletion in DM	Integration site in potato reference genome *S. tuberosum* Solyntus	Deletion in *S. tuberosum* Solyntus	Allele/gene interruption
**Sha.259**		chr08 50639935.50640103	168	chr08: 61597046.61597214	168	Yes
**Sha.6**		chr01: 80991615/80991907.80991631	15 or 275	chr01: 46645309/46645601.46645324	14 or 276	No
**Tig.261**		chr06: 51745278.51748279	3,001	chr06:57784008.57787380	3372	No
**Tig.266**		chr05: 1833125.1830115; vector	3,010	chr05: 1933912.1931048; vector	2864	No
**Vic.1**		chr03: 51680790.51683398	2,608	chr03: 54834739.54837347	2608	Yes
**Vic.14**		chr10: 58089463.58090381	918	chr10: 41942273.41943191	918	Yes
**Vic.26**		chr12: 50392051.50392106	55	chr12: 23573380.23573435	55	Yes
**Vic.39**		chr04: 58190658.58181618	9,040	chr04: 61026013.61035015	8998	Yes
**Vic.60**		chr05: 1172041.1172270	229	chr05: 1242483.1242724	241	Yes
**Vic.172**		chr06: 49081942.49082040	98	chr06: 55135926.55136032	106	No
**Vic.185**		chr09:60175647.60175823	176	chr09: 63093273.63093445	172	No
**Des.49**		chr08: 44263857.	ukn	chr08: 42614631.	ukn	Yes
**Sha.229**		chr03: 53209316.	ukn	chr03: 56299533.	ukn	No
**Sha.248**		chr09: 5523445.	ukn	chr09: 5768330.	ukn	No
**Sha.277**		chr07: 51084960/51083683.	ukn	chr07: 32416144.	ukn	No
**Tig.254**		chr03: 54912293.	ukn	chr03: 56552936.	ukn	No
**Vic.20**		chr03: 52038108.	ukn	chr03: 55147505.	ukn	No
**Vic.179**		chr05: 52195413.	ukn	chr05: 55400070.	ukn	No
**Vic.190**		chr09: 58446392.	ukn	chr09: 61421655.	ukn	No

The T-DNA integration position, as well as any deletions or existing alleles affected information remain unknown in events Des.52, Sha.2, Sha.102, Sha.105, Sha.210, Sha.271, Tig.6, Tig.267, Tig.999, Vic.2, Vic.12, Vic.153, Vic.168, Vic.169, and Vic.186. ‘ukn’ stands for unknown.

Out of the 34 transgenic events, 19 have a clearly identified insertion site in the potato reference genomes with at least one of its flanks. Eleven of them have a fully identified position from both flanks though one has two left flanks at the same site, and one has a short integration of vector sequence. For these 11, the deletion due to the insertion ranges from 14 bp to 8,998 bp in the *S. tuberosum* cultivar *Solyntus* reference genome. Of the 10 transgenic events with full T-DNA integration site known, 6 insertions have interrupted an existing gene or allele. Eight transgenic events have their insertion site identified partially by one of the two flanks (**Table 1**). Finally, 15 transgenic events have a complex insertion of their T-DNA, with T-DNA fragments duplicated including for some vector sequences, making their map position unclear.

**Figure 4:**
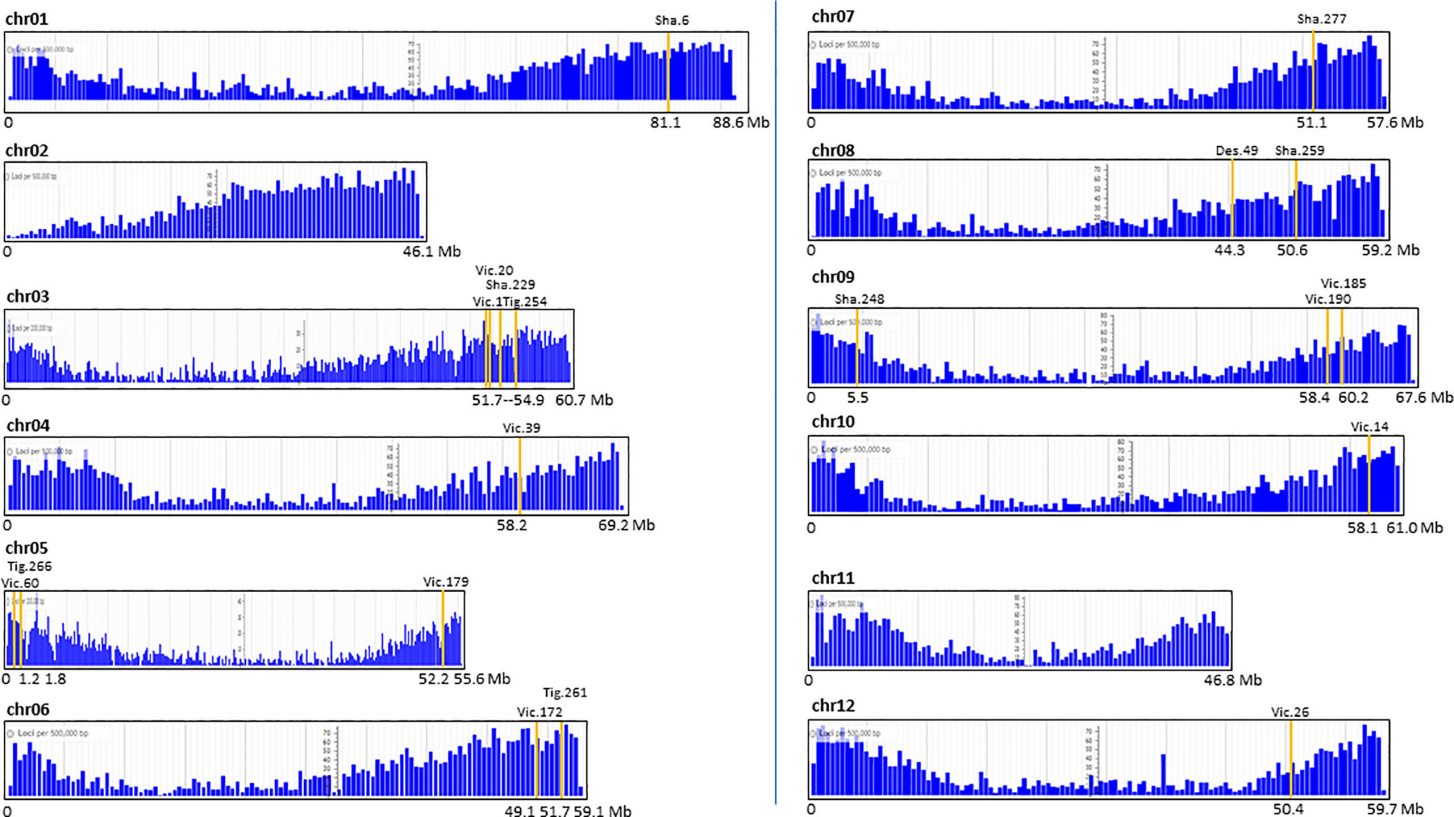
Map position of the single T-DNA insertion of 19 potato transgenic events on the 12 chromosomal gene-maps of the potato reference genome *S. tuberosum* Group Phureja DM1-3. The Y axis represents the number of loci (genes) per 0.5 Mb except for chr02, 05, and 11 where it is per 0.2 Mb. The X axis represents the chr length in Mb.

The map positions of the T-DNA in 19 transgenic events were placed on the potato reference genome and related to gene density. Interestingly, all insertions were in chromosome arms within gene-dense regions (**Figure 4**).

## Discussion

T-DNA insertion site characterization of transgenic events is still a challenging task which is usually carried out long after identifying stable transgenic events by techniques such as PCR, RTqPCR or Southern blot (Guttikonda et al., 2016). Transgenic events containing a single and intact copy of the T-DNA are more desirable as they are more likely to exhibit stable transgene expression, and Mendelian segregation behavior, than plants with multiple inserted T-DNA (OECD, 2010). T-DNA insertions can sometimes be accompanied by chromosomal rearrangements, translocations, incorporation of bacterial vector backbone sequence, T-DNA duplications and inversions, and other T-DNA sequence modifications that cannot be easily identified by the above-mentioned molecular techniques (Pucker et al., 2021).

In this study, we explored target capture sequencing (TCS) approach to characterize the insertion sites of T-DNA in 34 transgenic events of potatoes. Our method is similar to Southern-by-Sequencing but differs from it in that the probe used for capturing the target sequences is made from the entire T-DNA of the binary vector used in the transformation and not from a pool of plasmid vectors. This T-DNA-based probe increases the sequence depth of only this target and flanking regions, reducing the amount of sequencing data needed and simplifies the bioinformatic pipeline to usingexisting bioinformatics software’s. It requires though that the transgenic events are pre-selected to eliminate those with backbone vector sequences by PCR and multicopy by Southern blot. WGS such as the method of Kovalic et al. (2012) offers to by-pass this pre-screening and allow to identify insertions of fragments of backbone vector sequences which would not be detected by PCR screening. However, the sequencing cost and the bioinformatic complexity of WGS for complex genomes like potato are still much higher than our method of using pre-screening of backbone-vector-free and single T-DNA copy transgenic events, and TCS.

TCS generated 300 bp paired-end reads which mostly mapped to the T-DNA of the pCIP99 binary vector, which confirms the enrichment in the target locus. The coverage of the T-DNA was enough to identify unambiguously the T-DNA sequence of all 34 transgenic events. However, the coverage was uneven across the T-DNA sequence, with specific regions of the T-DNA with high coverage due to the presence of *R* gene homologs in the potato genome that were captured as well. In an unpublished study on the R protein homology to allergens and toxins, we detected 38 sequences from the potato reference genome which are homologous to RB, 35 sequences for RBLB2, and 18 sequences for RVNT1 as defined by any protein with 40% or greater amino acid sequence identity. In these regions, sequence analyses were conducted at higher sequence identity to segregate the reads specific to the T-DNA from those pertaining to the homologous sequences. This difficulty of ‘contaminating’ homologous gene sequences from the host genome can hardly be overcome when using PCR or Southern blotting.

The end of the inserted T-DNA was mapped by identifying the breakpoint where the homologous consensus sequence to the pCIP99 sequence stops. T-DNA breakpoints were identified for most of the inserted T-DNA. However, in about 10% (4 left and 3 right out of the 34) these could not be identified due to additional T-DNA fragments, fragmentation of the T-DNA, long integration of vector sequence, or recombination within regions of the *Rpi-blb2* gene with high similarity to its homologs in the potato genome. In these cases, WGS methods can be used to resolve these complex insertions.

The position of the breakpoints along the T-DNA was different between the left and right ends. The left junction was identified in a 330 bp region between the left border sequence and the *nptII* CDS, whereas the breakpoints in the right end were localized in a small region of 60 bp downstream of the right border sequence. This is consistent with the current model of integration of T-DNA into plant genomes, in which the first right end next to the right border (usually protected by VirD2 protein from exonuclease degradation) ligating to the plant DNA (Singer, 2018). However, the positions of the breakpoints differ from those identified in a collection of T-DNA insertion mutants from *Arabidopsis thaliana* which were localized mainly within the border sequences (Kleinboelting et al., 2015). Those authors used a different left border sequence, which is likely responsible for the more precise recombination. Although it is not a regulatory requirement, a more precise cut on the left end may be desirable, which can be obtained by changing the left border sequence of our binary plasmid with the one used for the *A. thaliana* insertion lines.

The flanking sequences were over 300 bp in more than 50% of the cases. These long flanks, which could extendup to 1.5 kb length, were made of reads without T-DNA sequence. In only a few transgenic events, (up to 3) for the left flank and 7 for the right flank out of 34 transgenic events, there were no consensus sequences overlapping the junctions between the T-DNA and the potato genome. Genome walking from near sequence tag or nested PCR amplicon sequencing has been successfully used to identify junction regions, but the length of the junction sequence is usually relatively short (Kalendar et al., 2021). This reduces the possibility of mapping T-DNA insertions in hosts with complex genomes, or those with limited available reference genomes, such as potato.

The integrity of the T-DNA was maintained for more than two thirds of the T-DNA insertions. The most frequent sequence divergence was the recombination of the right end within the *Rpi-blb2* gene, which is problematic as it changes the amino acid sequence and may affect the performance of the R protein. Only two T-DNA sequences had short sequence changes, including one SNP and one deletion, indicating that it is possible for these changes to occur but at a relatively low frequency. This result highlights the importance of verifying the sequence integrity of the T-DNA, and thus the transgenes in the transgenic events, and justify these criteria as a regulatory requirement.

The flanks were identified as potato genome (Tbr), T-DNA, or vector sequences and combinations thereof. The mapping of the Tbr left and right flanks were conducted using the reference genome *S. tuberosum* Group Phureja DM1-3 and the sequenced potato genome *S. tuberosum* cultivar *Solyntus* (Accession NC_008096). About two thirds of the transgenic events (19 out of 34) had their T-DNA localized by at least one of their flanks. Less than half of these events (7) were interrupting an existing gene. This high frequency of gene interruption is supported in earlier publications of preferential T-DNA mapping into the gene-dense regions of the chromosomes (Shilo et al., 2017). The pre-selection of the transgenic events of this study on kanamycin media and resistance to late blight disease favored the selection of transgenic events with T-DNA insertions in such regions. The insertions led to deletion of between 55 bp to 9,040 bp with, in few cases, local rearrangement of the potato genome sequence that has been observed previously (Kleinboelting et al., 2015).

## Conclusion

Our study provides evidence that T-DNA insertion characterization of a sizable number of transgenic events from any crop can be achieved using TCS combined with a simple bioinformatics pipeline, and that it does not require the extensive storage and bioinformatic skills that WGS does. TCS analyses accurately identified the T-DNA sequence, the precise breakpoints between the T-DNA ends and the potato flanking sequences, small deletions, single nucleotide polymorphisms, and rearrangements of both the introduced and flanking DNA sequences. Our results on 34 transgenic events indicate that approximately one-third of the transgenic events have a clean T-DNA insertion, with half of them interrupting an existing gene. For current commercial potato varieties, gene interruption may be compensated by other alleles, considering that only one gene out of 10 is a single copy (Sun et al., 2019). It should be noted that not all of the other transgenic events disqualify for future regulatory approval. Indeed, 12 transgenic events had an intact T-DNA and an extra partial copy of the T-DNA or vector sequences (discounting those with functional bacterial genes). In such cases of complex T-DNA insertions, TCS might need to be complemented by either long read sequencing methods or WGS, though that would significantly increase the cost of the experiments considering potato is a tetraploid crop. However, complex T-DNA insertions may trigger concerns over transgene silencing and their stability.

## Data availability statement

The data presented in the study are deposited in the NCBI Trace Archive Sequence Read Archive (SRA) repository, accession number PRJNA991282.

## Author contributions

EM prepared DNA and ran the technical experiments under HL guidance, did the bioinformatics analysis, and wrote the draft manuscript; HL designed and executed NGS library prep, and targeted capture enrichment and sequencing; AT designed the probes for target capture of the T-DNA regions; SZ supervised activities at Tennessee State University for the two USDA-FAS funded projects; MG supervised activities at the International Potato Center and, with EM, did the bioinformatics analyses and wrote the draft manuscript. All authors contributed to the article and approved the submitted version.
