# Supplementary material for: Identification of T-DNA structure and insertion site in transgenic crops using targeted capture sequencing
Source: Front Plant Sci. 2023 Jul 12;14:1156665. doi: 10.3389/fpls.2023.1156665 (PMC10369180; doi:10.3389/fpls.2023.1156665)
Supplement: Supplementary file 1 [file Table_1.docx]

Supplementary **Table 1**: Target capture sequencing data from 34 potato transgenic events, paired reads, and their coverage at 90% and 97% sequence identity. Q20 is a quality score in percentage of bases with sequencing error rate less than 1%.

|  | **Transgenic event** | **Paired reads** | **Q20** | **Reads mapped to T-DNA at 90% seq. id.** | **Coverage at 90% seq. id.*** | **Reads mapped to T-DNA at 97% seq. id.** | | **Coverage at 97% seq. id.*** |
| --- | --- | --- | --- | --- | --- | --- | --- | --- |
| 1 | **Des.49** | 1,027,254 | 89% | 803,875 | 12,976 | | 145,817 | 2,354 |
| 2 | **Des.52** | 1,338,469 | 89% | 1,041,833 | 16,817 | | 187,596 | 3,028 |
| 3 | **Sha.2** | 2,735,631 | 89% | 2,195,830 | 35,445 | | 624,344 | 10,078 |
| 4 | **Sha.6** | 2,474,894 | 79% | 2,155,110 | 34,788 | | 558,042 | 9,008 |
| 5 | **Sha.102** | 1,476,854 | 84% | 1,232,242 | 19,891 | | 403,550 | 6,514 |
| 6 | **Sha.105** | 1,687,581 | 94% | 1,543,765 | 24,920 | | 458,730 | 7,405 |
| 7 | **Sha.210** | 1,346,628 | 95% | 1,222,855 | 19,739 | | 588,006 | 9,492 |
| 8 | **Sha.229** | 702,144 | 79% | 452,858 | 7,310 | | 78,885 | 1,273 |
| 9 | **Sha.248** | 1,253,331 | 91% | 1,013,909 | 16,367 | | 198,979 | 3,212 |
| 10 | **Sha.259** | 1,320,754 | 95% | 1,149,501 | 18,555 | | 289,667 | 4,676 |
| 11 | **Sha.271** | 1,399,996 | 95% | 1,231,417 | 19,878 | | 363,816 | 5,873 |
| 12 | **Sha.277** | 1,403,726 | 95% | 1,228,309 | 19,827 | | 320,170 | 5,168 |
| 13 | **Tig.6** | 1,069,790 | 88% | 884,161 | 14,272 | | 227,820 | 3,677 |
| 14 | **Tig.254** | 1,336,048 | 89% | 1,187,010 | 19,161 | | 347,826 | 5,615 |
| 15 | **Tig.261** | 1,431,971 | 90% | 1,260,175 | 20,342 | | 320,311 | 5,170 |
| 16 | **Tig.266** | 1,496,816 | 91% | 1,063,120 | 17,161 | | 222,021 | 3,584 |
| 17 | **Tig.267** | 973,617 | 90% | 906,947 | 14,640 | | 358,177 | 5,782 |
| 18 | **Tig.999** | 1,988,650 | 95% | 1,323,975 | 21,372 | | 333,671 | 5,386 |
| 19 | **Vic.1** | 1,154,773 | 79% | 939,696 | 15,169 | | 180,163 | 2,908 |
| 20 | **Vic.2** | 1,406,997 | 90% | 1,268,196 | 20,471 | | 412,864 | 6,664 |
| 21 | **Vic.12** | 1,253,278 | 90% | 1,039,877 | 16,786 | | 269,723 | 4,354 |
| 22 | **Vic.14** | 1,206,173 | 90% | 1,000,351 | 16,148 | | 240,610 | 3,884 |
| 23 | **Vic.20** | 1,090,124 | 89% | 892,360 | 14,405 | | 264,875 | 4,276 |
| 24 | **Vic.26** | 1,525,303 | 95% | 1,373,398 | 22,169 | | 436,805 | 7,051 |
| 25 | **Vic.39** | 1,276,038 | 91% | 1,023,948 | 16,529 | | 199,140 | 3,215 |
| 26 | **Vic.60** | 1,397,337 | 95% | 1,077,701 | 17,396 | | 197,174 | 3,183 |
| 27 | **Vic.153** | 1,302,087 | 95% | 1,007,431 | 16,262 | | 224,339 | 3,621 |
| 28 | **Vic.168** | 1,542,606 | 94% | 1,386,727 | 22,385 | | 531,072 | 8,573 |
| 29 | **Vic.169** | 1,506,551 | 95% | 1,318,082 | 21,277 | | 342,363 | 5,526 |
| 30 | **Vic.172** | 1,531,539 | 95% | 1,355,735 | 21,884 | | 366,547 | 5,917 |
| 31 | **Vic.179** | 1,090,124 | 95% | 1,410,774 | 22,773 | | 472,658 | 7,630 |
| 32 | **Vic.185** | 1,430,650 | 95% | 1,294,006 | 20,888 | | 343,072 | 5,538 |
| 33 | **Vic.186** | 1,513,160 | 95% | 1,340,142 | 21,633 | | 392,465 | 6,335 |
| 34 | **Vic.190** | 1,355,544 | 95% | 1,185,605 | 19,138 | | 343,789 | 5,549 |
|  | **Min** | **702,144** | **79%** | **452,858** | **7,310** | | **78,885** | **1,273** |
|  | **Max** | **2,735,631** | **95%** | **2,195,830** | **35,445** | | **624,344** | **10,078** |
|  | **Average** | **1,413,131** | **91%** | **1,200,321** | **19,376** | | **330,738** | **5,339** |

Supplementary **Table 2**: T-DNA insert characterization from left to right end including breakpoint in the junction of the T-DNA with flanks, length and nature of the flanks, and integrity of the inserted T-DNA sequence. ‘Tbr’ stands for potato genome, f(X) for filler DNA of X nucleotide, and ‘ukn’ for unknown. The first group of transgenic events from Des.52 to Vic.185 are those with T-DNA structure intact and no extra fragment of T-DNA inserted; the second group from Sha.6 to Sha.210 are those with T-DNA structure intact and at least one extra fragment of T-DNA inserted; and the last group of transgenic events from Vic.12 to Tig.266 have either additional vector sequence or altered T-DNA.

| **Transgenic event** | **Left breakpoint(s)** | **Left flank lengths (bp)** | **Left flanks nature** | **Right breakpoint(s)** | **Right flank lengths (bp)** | **Right flanks nature** | **T-DNA structure** |
| --- | --- | --- | --- | --- | --- | --- | --- |
| **Des.52** | 6,370 | 295 | Tbr | 24,658 | 484 | Tbr | Intact |
| **Sha.105** | 6,503 | 213 | Tbr+f(48) | 24,687 | 50 | Tbr | Intact |
| **Sha.259** | 6,248 | 616 | Tbr | 24,714 | 699 | Tbr | Intact |
| **Tig.254** | 6,279 | 664 | Tbr+f(48)) | 24,715 | 483 | f(25)+Tbr | Intact |
| **Vic.2** | 6,177 | 631 | Tbr | 24679,24711 | 300; 767 | ukn; f(40)+Tbr Tbr | Intact |
| **Vic.39** | 6,481 | 474 | Tbr | 24,710 | 446 | Tbr | Intact |
| **Vic.60** | 6,180 | 395 | Tbr | 24,714 | 451 | Tbr | Intact |
| **Vic.172** | 6,185 | 536 | Tbr | 24,712 | 644 | Tbr | Intact |
| **Vic.179** | 6,289 | 409 | Tbr | 24,715 | 327 | Tbr | Intact |
| **Vic.185** | 6,338 | 94 | Tbr | 24,709 | 327 | Tbr | Intact |
| **Sha.6** | 6,427; 6,458 | 751; 323 | Tbr,T-DNA | 24,715 | 262 | Tbr | Intact |
| **Sha.271** | 6,206; 6,254; 6,356 | ukn | Tbr; T-DNA | 24,669 | ukn | T-DNA | Intact |
| **Sha.277** | 6182; 6,182 | 736; 860 | Tbr; T-DNA | 24,600 | ukn | T-DNA | Intact |
| **Tig.6** | 6,215 | 274 | T-DNA | 24707,24710,24717 | ukn | Tbr,T-DNA | Intact |
| **Tig.999** | 6,177 | 507 | Tbr | 24,712 | 227 | Tbr+T-DNA | Intact |
| **Vic.14** | 6,364 | 469 | Tbr | 18,967; 24,715 | 486; 298 | f(23)+T-DNA; Tbr | Intact |
| **Vic.26** | 6,464 | 695 | Tbr | 24695,24701 | ukn | T-DNA | Intact |
| **Vic.153** | 6,468 | 1,069 | T-DNA | 24,713 | 443 | T-DNA | Intact |
| **Vic.169** | 6,149 | 374 | Tbr; T-DNA | 24,715 | 374 | Tbr,T-DNA | Intact |
| **Vic.190** | 6,296 | 495 | Tbr | 24,713 | 1,354 | T-DNA | Intact |
| **Sha.210** | 6,339 | 528 | Tbr | 24,675; 24,682; 24,710; 24,712; 24,715 | 283; 322; 394; 322; 475 | Tbr; f(20)+Vector | Intact |
| **Vic.12** | ukn | 1,159 | Run-off Vector | 24703, | 510 | T-DNA | Intact |
| **Vic.168** | ukn | 1,380 | Run-off Vector | 24,672; 24708,24711 | 310; 303; 282 | Tbr+T-DNA; T-DNA+f(14); T-DNA+f(6) | Intact |
| **Vic.186** | ukn | 1,337 | Run-off Vector | 24,696 | 544 | T-DNA | Intact |
| **Vic.20** | 6,340 | 396 | Tbr | 24,709 | 501 | Ti-plasmid | Intact |
| **Sha.102** | ukn | ukn | ukn | ukn | ukn | ukn | Fragmented |
| **Tig.267** | 6,436 | 282 | Tbr+T-DNA | 24715; 24715 | 299; 331 | Tbr; f(23); Vector | Fragmented |
| **Sha.2** | 6,444 | 895 | Tbr | 24,130 | 796 | Tbr | Truncated in Rpi-blb2 |
| **Sha.229** | 6,246 | 371 | Tbr | ukn | ukn | ukn | Truncated in Rpi-blb2 |
| **Sha.248** | 6,342 | 648 | Tbr | ukn | ukn | ukn | Truncated in Rpi-blb2 |
| **Tig.261** | 6,342 | 543 | Tbr | 21,967 | 481 | Tbr | Truncated in Rpi-blb2 |
| **Vic.1** | 6,363 | 404 | Tbr+f(8) | 23,967 | 231 | f(8); Tbr | Truncated in Rpi-blb2 |
| **Des.49** | 6,284 | 555 | Tbr | 24,711 | 585 | Tbr | SNP at 17,365 |
| **Tig.266** | 6417; ukn | 393; ukn | Tbr; Run-off Vector | 24,673 | 193 | Tbr | Del [21,058-21080] |
